# MultiVirusConsensus: an accurate and efficient open-source pipeline for identification and consensus sequence generation of multiple viruses from mixed samples

**DOI:** 10.1093/bioadv/vbag256

**Published:** 2026-09-02

**Authors:** Niema Moshiri

**Affiliations:** Department of Computer Science & Engineering, UC San Diego, San Diego, CA 92093, United States

## Abstract

**Motivation:**

Viral surveillance from mixed samples (e.g. wastewater) has become critical in public health efforts to track and contain pathogens. However, existing open-source bioinformatics tools for viral consensus sequence generation are optimized for individual viruses (rather than multiple potential viruses of interest).

**Results:**

MultiVirusConsensus (MVC) is an accurate and efficient open-source pipeline for identification and consensus sequence generation of multiple viruses from mixed samples. It utilizes the memory-efficient ViralConsensus tool to simultaneously perform consensus sequence calling on all viruses of interest (1) completely in parallel, and (2) by piping datastreams between tools without writing/reading intermediate files (thus eliminating slowdowns related to slow disk accesses).

**Availability:**

MultiVirusConsensus (MVC) is freely available as an open-source software project at: https://github.com/niemasd/MultiVirusConsensus

## 1 Introduction

The epidemiological applications of viral sequencing have expanded rapidly as a result of the COVID-19 pandemic. The real-time reconstruction of viral genomes collected from individual patients (Moshiri 2023, Ji *et al.* 2024) as well as mixed wastewater samples (Karthikeyan *et al.* 2022) are able to inform real-time public health intervention (Oude Munnink *et al.* 2021, Fielding-Miller *et al.* 2023, Matteson *et al.* 2023, Keehner *et al.* 2024, Wertheim *et al.* 2025). While the majority of recent efforts in this space have focused on a single virus of interest at a time, recent innovations such as the Illumina Viral Surveillance Panel have enabled the simultaneous sequencing of multiple viruses of interest (Shamblin *et al.* 2026).

The reconstruction of consensus genome sequences from raw sequence data requires the use of various bioinformatics pipelines such as iVar (Grubaugh *et al*. 2019), HAVoC (Truong Nguyen *et al*. 2021), V-pipe (Posada-Céspedes *et al*. 2021), ViReflow (Moshiri *et al*. 2022), and many more. However, to our knowledge, all existing open-source viral genome reconstruction pipelines are intended to reconstruct the genome of a *single* virus of interest in a single run, and the only existing pipeline for reconstructing the genomes of *multiple* viruses of interest in a single run is Illumina’s commercial BaseSpace “DRAGEN Microbial Enrichment Plus App” that accompanies their Viral Surveillance Panel.

Here, we introduce MultiVirusConsensus (MVC), an accurate and efficient open-source pipeline for identification and consensus sequence generation of multiple viruses from mixed samples. In order to demonstrate its utility, we benchmark MVC on multiple real datasets and on a synthetic dataset, and we compare it to existing best-practice methods.

## 2 Results and discussion

MultiVirusConsensus (MVC) is a command-line tool written in Python that requires Minimap2 (Li 2018), Samtools (Li *et al.* 2009), and ViralConsensus (Moshiri 2023). MVC takes the following as required input: (i) one or more FASTQ, SAM, or BAM files containing the reads, (ii) one or more FASTA files containing one or more viral reference genome sequences, and (iii) an output directory to write the results. Optionally, the user can also provide the following: (i) one or more BED files containing amplicon sequencing primers with respect to the provided reference genome sequences for optional primer trimming, (ii) the maximum number of threads to use (default: max), (iii) how to handle multimapped reads (“all” to keep all mappings, “best” to keep only the single best mapping, or “none” to discard multimapped reads; default: “none”), and (iv) the paths to all dependency executables (default: assume they’re in the user’s PATH).

**Figure 1:**
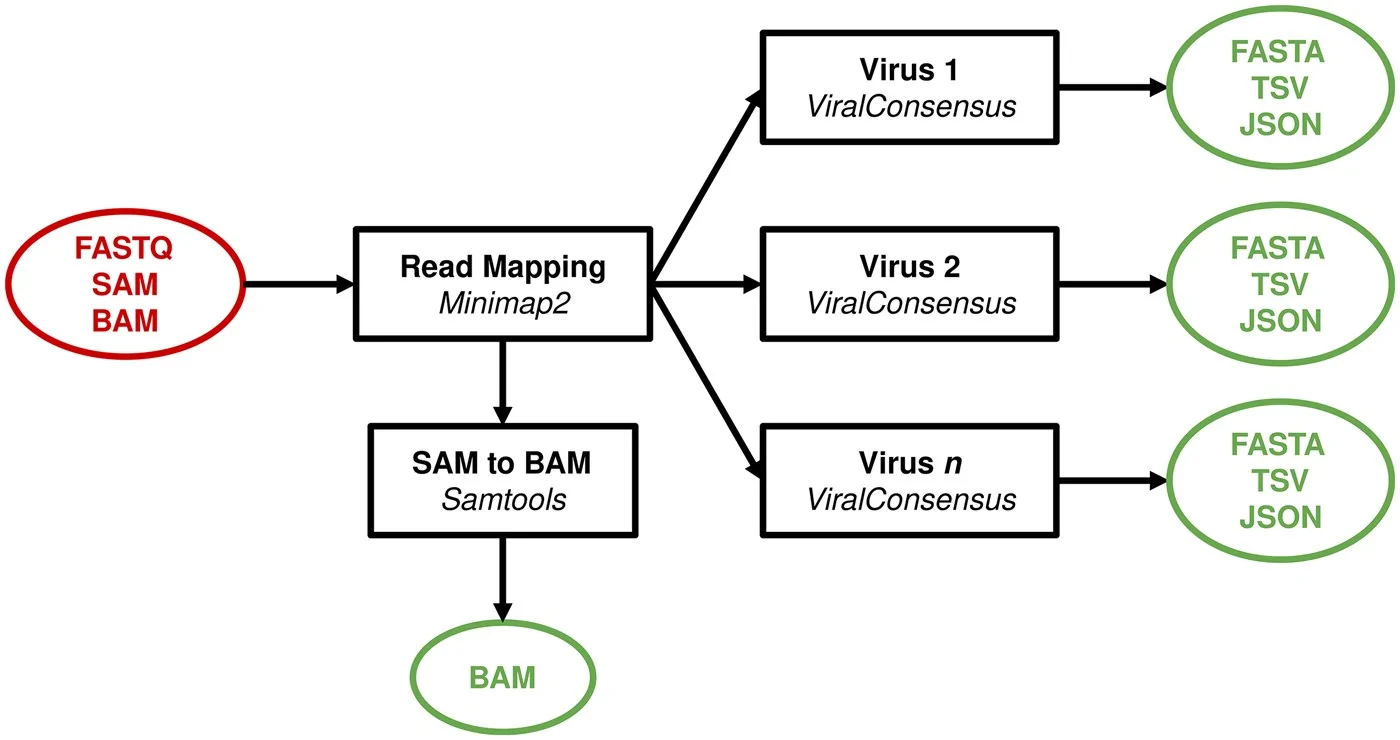
MultiVirusConsensus (MVC) pipeline. Diagram depicting the MVC pipeline. FASTQ files are fed to Minimap2 (for read mapping; SAM/BAM input are passed through without remapping), then simultaneously fed to Samtools (for writing a single merged BAM) as well as to a separate ViralConsensus process per viral reference genome sequence (for consensus sequence calling).

The MVC pipeline is visualized in Fig. 1. First, the reference genomes are loaded from the input FASTA files, and they are merged into a single FASTA file (for use during read mapping) as well as into separate FASTA files with a single reference sequence per file (for use during consensus sequence calling). Next, if provided, the primer BED files are loaded and written into separate BED files with a single reference genome’s primers per file. Then, the actual pipeline is executed: (i) the FASTQ reads are mapped to the merged reference genome FASTA using Minimap2 (SAM/BAM inputs are not remapped), (ii) the read mapping results are written to a single merged BAM file using Samtools, and (iii) the reads are separated into individual consensus sequence calling operations (one per viral reference genome sequence) using ViralConsensus.

Importantly, MVC takes a “single-pass” approach to the multi-virus consensus calling pipeline. First, in the read mapping step of the pipeline, MVC performs a single pass over the FASTQ input by executing a single Minimap2 process on a single merged FASTA file containing all of the reference sequences (rather than mapping the entire FASTQ input repeatedly for each reference sequence). Second, in the consensus calling step of the pipeline, MVC performs a single pass over Minimap2’s SAM output by feeding each alignment directly to the ViralConsensus process corresponding to its reference sequence (rather than parsing the entire SAM output repeatedly for each reference sequence).

Importantly, in order to avoid writing and subsequently reading intermediate files at each compute-intensive step, the reads are loaded from disk at the beginning of the pipeline, and aside from small reference genome files (KBs per file), all subsequent data movement occurs directly between processes via pipes, until output files are ultimately written to disk at the ends of the pipeline.

**Figure 2:**
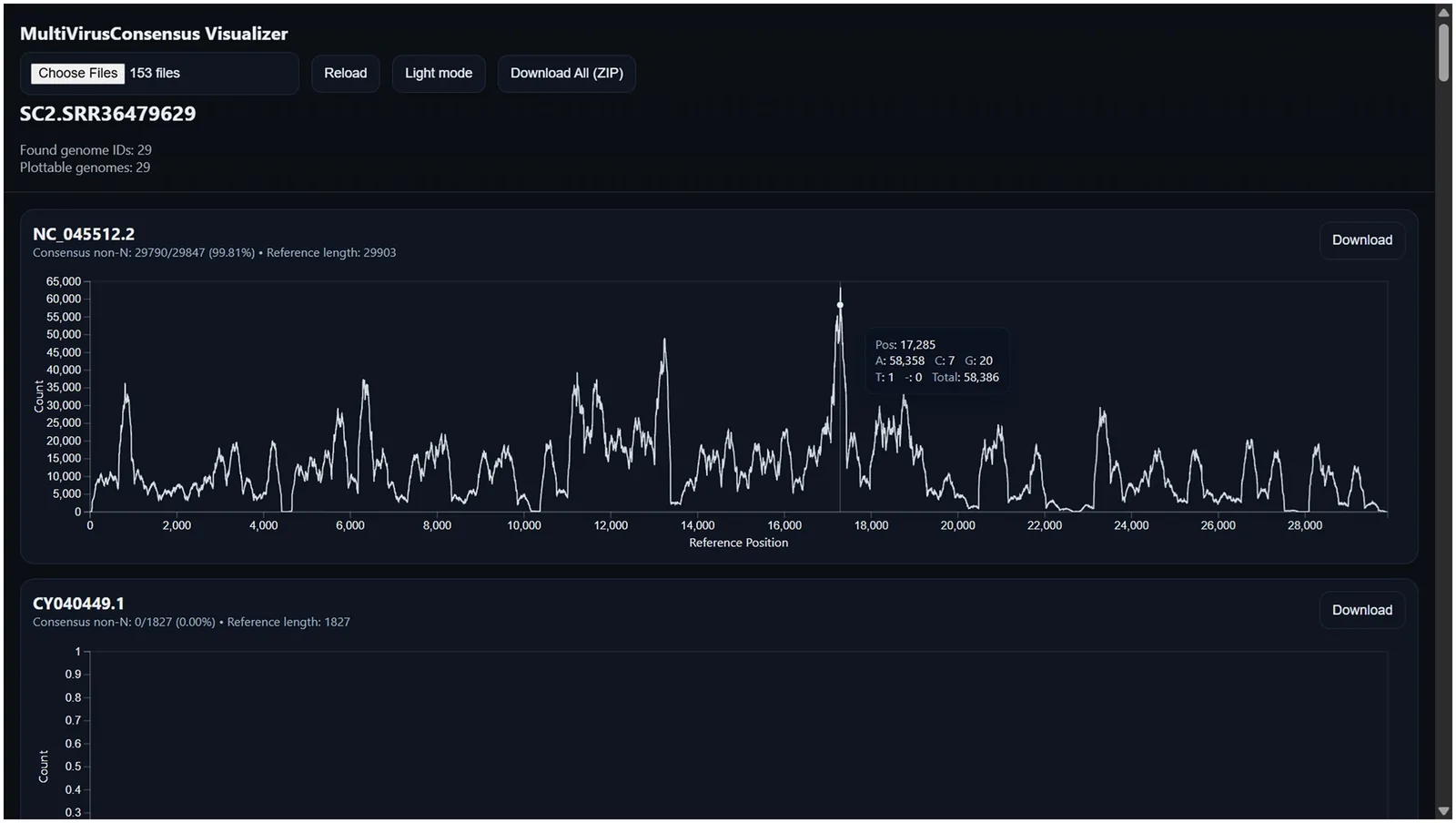
MultiVirusConsensus (MVC) companion web application. Screenshot demonstrating the MVC companion web application. The screenshot shows the web application visualizing the results from running MVC on a real SARS-CoV-2 sequencing dataset: as expected, the top (highest-completeness) consensus genome is SARS-CoV-2, which is the only coverage plot showing significant coverage.

In order to assist users as they interpret the results produced by MVC, we also provide a companion web application: https://niema.net/MultiVirusConsensus (Fig. 2). Users can simply select an MVC output folder, and the web application will load the results and produce interactive coverage plots that are sorted in descending order of consensus genome completeness (i.e. the number of unambiguous bases in the consensus genome divided by the length of the reference genome). Importantly, the user’s data are not sent anywhere external: the web application is fully client-side (and thus secure), and it can even be executed locally by simply downloading and opening its HTML file. This is important for HIPAA compliance, as users who have not already host filtered their reads could accidentally include patient-identifiable information in the output BAM file (which includes reads that failed to map to the viral reference genome sequences and thus may contain reads from the host).

In order to benchmark MVC, we first built a reference sequence collection consisting of the following 8 reference genomes (29 sequences in total due to multi-segment Influenza genomes): CY040449.1–56.1 (Influenza B Segments 1–8), NC_007366.1–73.1 (Influenza A Segments 1–8), NC_026431.1–38.1 (Influenza A Segments 1–8 Coding Sequences), NC_045512.2 (SARS-CoV-2), OP890336.1 (RSV A), OP965707.1 (RSV B), NC_039199.1 (HMPV), and NC_023891.1 (HPV). We then evaluated the runtime, peak memory, and accuracy of MVC by processing one simulated and four real sequencing datasets. The simulated dataset was produced from the reference sequence collection by using ART version “MountRainier 2016–06-05” (Huang *et al*. 2012) to simulate Illumina HiSeq 2500 paired-end short reads at 1000× coverage. The real datasets were obtained by selecting four respiratory viruses with similar symptoms that are of typical interest to public health efforts (HMPV, Influenza A, RSV, and SARS-CoV-2) and searching the Sequence Read Archive (SRA) for Illumina MiSeq datasets containing just one of the viruses of interest: SRR8776440 (HMPV), SRR37308007 (Influenza A), SRR37377179 (RSV), and SRR36479629 (SARS-CoV-2).

We then ran MVC v0.1.0 using its default settings on all four real datasets, a “mixed” real dataset produced by concatenating the four real datasets, and the simulated dataset. For the real datasets, a read mapping was determined to be “correct” if it mapped to the virus already known to be in the sample, or “incorrect” if it mapped to any other reference genome in the collection. For the simulated dataset, a read mapping was determined to be “correct” if it mapped to the reference sequence from which it was simulated, or “incorrect” if it mapped to any other reference sequence in the collection. All datasets and their respective outputs can be found in the following GitHub repository: https://github.com/niemasd/MultiVirusConsensus-Paper.

Note that the HMPV and SARS-CoV-2 datasets were produced via amplicon sequencing (but were already primer-trimmed), whereas the Influenza A and RSV datasets were not. We expect non-amplicon datasets to have lower proportions of their reads map successfully: accuracy should be assessed by comparing the ratio of the number of correctly-mapped reads vs. the number of incorrectly-mapped reads.

As can be seen in Table 1, across all datasets, the number of reads that map to the correct reference sequence is orders of magnitude larger than the number of reads that map to an incorrect reference sequence, especially in amplicon sequencing datasets. Importantly, this holds even in mixed samples (both real and simulated).

**Table 1:** MultiVirusConsensus (MVC) benchmark results.

	Fragments	Correct	Incorrect	Time (s)	Mem (KB)
**HMPV**	481 334	221 076	363	12.55	417 880
**Flu A**	682 307	59 212	34	6.44	433 684
**RSV**	648 771	118 914	45	9.34	430 124
**SC2**	1 315 962	1 309 009	0	32.43	642 560
**Mix**	3 128 374	1 708 211	442	58.26	645 396
**Sim**	398 500	398 465	0	9.53	446 208

Number of fragments, number of correctly mapped fragments, number of incorrectly mapped fragments, runtime (in seconds), and peak memory usage (in kilobytes) for the four real datasets (HMPV, Influenza A, RSV, and SARS-CoV-2), the “mixed” real dataset, and the simulated dataset. All benchmarks were run on a 2.8 GHz Intel i7-1165G7 CPU with 8 cores and 16 GB of memory. Fragments were only considered “Correct” or “Incorrect” if they were “perfect pairs” (i.e. both reads in a pair mapped to the same reference sequence in the correct orientation and spacing).

The runtime, which scales roughly linearly with respect to sequencing dataset size and is roughly constant with respect to reference genome collection size, ranged between 21 seconds and 4 minutes despite extremely high coverage in the datasets.

The peak memory usage, which should scale roughly linearly with respect to reference genome collection size and which should be roughly constant with respect to sequencing dataset size, ranged between 417–645 MB. The fact that there was any variation in the peak memory usage despite using the same reference genome collection is surprising, as the largest memory usage should be the result of having a separate ViralConsensus run for each reference genome running in parallel (and the peak memory usage of ViralConsensus is linear with respect to reference genome length due to the memory allocation of counters for each reference position). The most likely reason for the ∼228 MB variation in peak memory usage across the runs, despite using the same reference genome sequences, can likely be attributed to many of the ViralConsensus runs terminating very quickly (potentially before others spawn) when the sequencing dataset size is relatively small (where “small” is actually larger than 1000X coverage). Nevertheless, in all benchmarks, MVC ran with a peak memory usage far lower than 1 GB, meaning this dataset can be comfortably executed on lightweight devices (e.g. a Raspberry Pi).

In order to demonstrate the scalability and accuracy of MVC in the context of existing best practice tools, paired-end short reads were simulated from a collection of 548 reference sequences according to the same process as previously mentioned, but at 10×, 20×, and 30× coverage, with 5 replicates for each coverage. The complete collection of reference sequences can be found in the following GitHub repository: https://github.com/niemasd/MultiVirusConsensus-Paper.

We then ran ViralConsensus v1.0.4 and iVar Consensus v1.4.4 on each simulated dataset sequentially for each reference sequence, and we ran MVC v0.1.3 in multimapped “all” mode on each simulated dataset using the complete reference collection. Illumina’s BaseSpace “DRAGEN Microbial Enrichment Plus App” was excluded from the pipeline comparison benchmark because the authors do not have access to it (as it is commercial software). Accuracy was measured for each consensus sequence by calculating its percent identity vs. its respective reference sequence.

**Figure 3:**
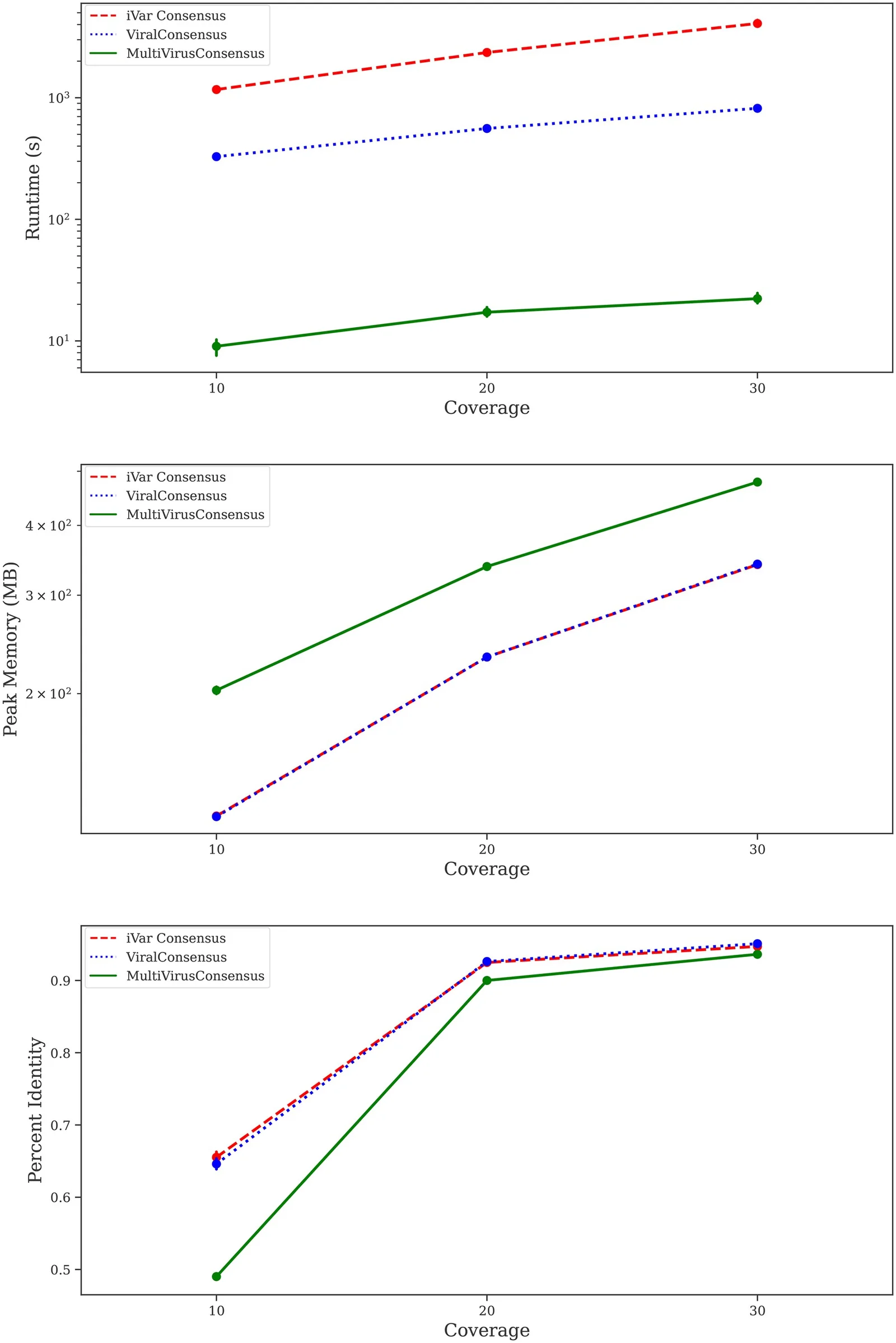
Pipeline comparison benchmark results. Runtime (top), peak memory usage (middle), and consensus sequence percent identity vs. corresponding reference (bottom) of iVar Consensus, ViralConsensus, and MultiVirusConsensus (MVC). All benchmarks were run on a 2.8 GHz Intel i7-1165G7 CPU with 8 cores and 16 GB of memory. For iVar Consensus and ViralConsensus, the entire pipeline (including read mapping) was run sequentially on each viral reference sequence: the overall runtime was calculated as the sum of the individual per-reference runtimes, and the overall peak memory usage was calculated as the maximum of the individual per-reference peak memory usages. Note that MVC’s speedup is purely due to its “single-pass” approach.

As can be seen in Fig. 3, across all coverages, MVC was 1–2 orders of magnitude faster, but it had 1.5–2 times the peak memory usage. Rather than any algorithmic innovation, this speedup is purely due to MVC’s “single-pass” approach. MVC had consistently lower accuracy, but by 30× coverage, all pipelines had similarly high accuracy.

Also, to aid users who may be analyzing real datasets that may or may not have a mixture of multiple strains or lineages of the same virus, we have provided a helper script (check_mixture.py) that analyzes a MultiVirusConsensus output folder and attempts to predict if the sample contained a mixture of multiple strains/lineages for any of the provided reference sequences. Currently, this script performs a simple heuristic where, for each position count TSV in the ViralConsensus output, for each position in the TSV, if the coverage at the position is greater than or equal to a user-provided threshold (with a reasonable default provided) and the proportion of the most frequent base at the position is below a user-provided threshold (with a reasonable default provided), a warning message is displayed about the corresponding virus. This simple approach was able to successfully detect that the simulated multi-lineage dataset was indeed a mixture. In the future, this script can be made more sophisticated (e.g. by learning rules based on the base frequencies at all positions of the TSV, or by running on a BAM directly to perform per-read analyses that incorporate more complex statistical tests, etc.).

In sum, we introduce MultiVirusConsensus (MVC), an accurate and efficient open-source pipeline for identification and consensus sequence generation of multiple viruses from mixed samples. ViralConsensus is fast and memory-efficient and can even be executed with hundreds of viruses of interest on a laptop, making it an ideal tool for real-time viral molecular surveillance. We hope ViralConsensus will aid viral molecular epidemiologists in their efforts to analyze viral sequence data at massive scale, particularly as an open-source alternative to similar commercial pipelines such as Illumina’s BaseSpace “DRAGEN Microbial Enrichment Plus App” that accompanies their Viral Surveillance Panel.

## Data Availability

All code and data are accessible at https://github.com/niemasd/MultiVirusConsensus and https://github.com/niemasd/MultiVirusConsensus-Paper.
